# Notch signaling is activated in knee-innervating dorsal root ganglia in experimental models of osteoarthritis joint pain

**DOI:** 10.1186/s13075-023-03039-1

**Published:** 2023-04-15

**Authors:** Lai Wang, Shingo Ishihara, Jun Li, Rachel E. Miller, Anne-Marie Malfait

**Affiliations:** Division of Rheumatology, Department of Internal Medicine, Rush University Medical Center, 1611 West Harrison Street, Suite 510, Chicago, IL 60612 USA

**Keywords:** Notch signaling, Dorsal root ganglia, Osteoarthritis, Pain, Mouse model

## Abstract

**Background:**

We aimed to explore activation of the Notch signaling pathway in knee-innervating lumbar dorsal root ganglia (DRG) in the course of experimental osteoarthritis (OA) in mice, and its role in knee hyperalgesia.

**Methods:**

Cultured DRG cells were stimulated with the TLR4 agonist, lipopolysaccharide (LPS). Notch signaling in the cells was either inhibited with the γ-secretase inhibitor, DAPT, or with soluble Jagged1, or activated through immobilized Jagged1. CCL2 production was analyzed at mRNA and protein levels. In in vivo experiments, knee hyperalgesia was induced in naïve mice through intra-articular (IA) injection of LPS. The effect of inhibiting Notch signaling was examined by pre-injecting DAPT one hour before LPS. OA was induced through surgical destabilization of the medial meniscus (DMM) in male C57BL/6 mice. Gene expression in DRG was analyzed by qRT-PCR and RNAscope in situ hybridization. Activated Notch protein (NICD) expression in DRG was evaluated by ELISA and immunofluorescence staining. DAPT was injected IA 12 weeks *post* DMM to inhibit Notch signaling, followed by assessing knee hyperalgesia and CCL2 expression in the DRG.

**Results:**

In DRG cell cultures, LPS increased NICD in neuronal cells. Inhibition of Notch signaling with either DAPT or soluble Jagged1 attenuated LPS-induced increases of *Ccl2* mRNA and CCL2 protein. Conversely, activating Notch signaling with immobilized Jagged1 enhanced these LPS effects. In vivo, IA injection of LPS increased expression of Notch genes and NICD in the DRG. Pre-injection of DAPT prior to LPS alleviated LPS-induced knee hyperalgesia, and decreased LPS-induced CCL2 expression in the DRG. Notch signaling genes were differentially expressed in the DRG from late-stage experimental OA. *Notch1*, *Hes1*, and NICD were increased in the neuronal cell bodies in DRG after DMM surgery. IA administration of DAPT alleviated knee hyperalgesia *post* DMM, and decreased CCL2 expression in the DRG.

**Conclusions:**

These findings suggest a synergistic effect of Notch signaling with TLR4 in promoting CCL2 production and mediating knee hyperalgesia. Notch signaling is activated in knee-innervating lumbar DRG in mice with experimental OA, and is involved in mediating knee hyperalgesia. The pathway may therefore be explored as a target for alleviating OA pain.

**Supplementary Information:**

The online version contains supplementary material available at 10.1186/s13075-023-03039-1.

## Background

Our incomplete understanding of the mechanisms underlying joint pain in osteoarthritis (OA) accounts for the general ineffectiveness of analgesics and hampers the development of new pharmacological treatments [1]. The first step in pain generation is nociception, a process whereby noxious stimuli activate nociceptors, specialized sensory neurons that innervate peripheral tissues [2]. These stimuli generate action potentials that carry the pain signal from the innervated tissues to the cell bodies in the dorsal root ganglia (DRG). From there, the signal is relayed to the dorsal horn of the spinal cord, where nociceptors synapse with neurons in central pain pathways, and then travels further up to the brainstem and higher brain regions, where it is consciously perceived as pain.

The pain signal can be modulated—often amplified—at different stages along the pain pathway, including in the DRG, through neuronal crosstalk and interactions between neurons and non-neuronal cells [3]. All animal models of chronic pain are characterized by extensive changes in the DRG, including cellular, molecular, and biophysical changes. It can be expected that a detailed description of the precise nature of such DRG changes will deepen our understanding of mechanisms underlying pain and facilitate the identification of molecular targets. In neuropathic pain models, DRG changes have been extensively delineated [4, 5], and more recently, there has been increased characterization of the DRGs that contain the cell bodies of knee-innervating afferents in mouse models of OA [3, 6]. For example, in slowly progressive experimental OA induced by surgical destabilization of the medial meniscus (DMM) in the mouse knee, joint damage and the associated pain-related behaviors are accompanied by specific temporally regulated cellular and molecular changes in the lumbar L3-L5 DRG [3, 6].

A recent microarray analysis of the L3-L5 DRG at 4, 8, and 16 weeks after DMM revealed a strong regulation of innate neuro-immune pathways, especially in the later stages of the model, 8–16 weeks after DMM, when persistent pain is associated with severe joint damage [7]. Further analysis of these DRG microarray data revealed a clear regulation of genes encoding molecules in the Notch signaling pathway after DMM, also in the later stages of the model. Because this pathway has been extensively investigated in nervous system development [8], we decided to validate these findings, and explore if the Notch signaling pathway may play a role in knee joint pain.

Notch signaling is a cell-to-cell signaling pathway that plays a major regulatory role in cell fate and differentiation, including in the nervous system [9]. Canonical Notch signaling is mediated by Notch receptors, which are transmembrane receptors that are activated by the Delta-like (Dll)/Jagged (Jag)-family ligands presented by neighboring cells. Following ligand-receptor interactions, the intracellular domain of Notch receptors (Notch intracellular domain or NICD) is released from the membrane by a sequence of proteolytic cleavage events mediated by ADAM10/17 (a disintegrin and metalloprotease 10/17), and subsequently the γ-secretase complex. The NICD then translocates to the nucleus and binds to the DNA binding transcription factor, RBPJ (recombination signal binding protein for immunoglobulin kappa J region), and Mastermind-family coactivators, facilitating the transcription of Notch downstream target genes such as HES (hairy and enhancer of split) family genes [10].

An evolutionary conserved transcription factor cascade downstream of Notch signaling is necessary for both the maintenance of neural progenitor cell character and the progression of neurogenesis [11, 12]. Furthermore, links between Notch signaling and pain perception have been explored in rat models of peripheral neuropathic pain [13–15]. Therefore, we decided to investigate the Notch signaling pathway in experimental models of knee pain. Of interest, it has been reported that Notch signaling may regulate innate immunity and inflammation through a crosstalk with toll-like receptor (TLR) signaling [16]. Since we have previously reported that damage-associated molecular patterns (DAMPs) present in OA joints can excite nociceptors through neuronal TLR4 [17], and that the pro-algesic chemokine, CCL2, is released upon TLR activation and plays a key role in initiating and maintaining pain in the DMM model [17–19], we investigated the crosstalk between Notch signaling and TLR4 signaling in activating CCL2 production in the DRG and mediating knee hyperalgesia. We also explored whether Notch signaling is activated in the DRG of mice with experimental knee OA, and whether this activation contributes to knee hyperalgesia.

## Methods

### Animals and surgery

All animal experiments were approved by the Institutional Animal Care and Use Committee at Rush University Medical Center. A total of 220 male C57BL/6 J mice were used for these experiments, including 101 naïve mice and 119 mice that underwent DMM or sham surgery. DMM or sham surgery was performed in the right knee of 10-week old mice under general anesthesia using isoflurane, as previously described [18, 20]. Briefly, the medial meniscotibial ligament (MMTL) was transected after medial parapatellar arthrotomy and dissection of the anterior fat pad. Sham surgery was through the same approach without MMTL transection.

### DRG cell cultures

Bilateral L3-L5 DRG were collected from 10-week old male naïve C57BL/6 mice and pooled for enzymatic digestion using collagenase 4 and papain, as previously described [18]. A total of 36 mice were used. Dissociated DRG cells were plated on poly-L-lysine and laminin-coated glass coverslips in multi-well plates, and cultured in F12 medium supplemented with 1 × N2 and 0.5% fetal bovine serum.

On day 4, TLR4 signaling was stimulated by adding lipopolysaccharide (LPS) (Catalog# tlrl-3pelps, InvivoGen, San Diego, CA) at a concentration of 1 μg/mL. Notch signaling was inhibited either by a γ-secretase inhibitor, DAPT (*N*-[*N*-[3, 5-diflurophenylacetate]-l-alanyl]-(S)-phenylglycine t-butyl ester) (Catalog# 565784, Calbiochem, San Diego, CA), or by a soluble form of the Jagged1 (188–204) peptide (sJag1, CDDYYYGFGCNKFCRPR) (AnaSpec Inc., Fremont, CA). DAPT (25 μM) or sJag1 (40 μM) was added 1 h before LPS. Control groups were treated with vehicle (0.1% dimethyl sulfoxide, DMSO) and scrambled Jagged1 (RCGPDCFDNYGRYKYC, AnaSpec), respectively.

In another set of cultures, Notch signaling was induced by using immobilized Jagged1 ligand as described [21]. Briefly, Corning® BioCoat™ Poly-D-Lysine/Laminin 4-well chamber slides (Corning, Bedford, MA) were coated overnight with 50 μg/mL of protein G (Invitrogen), and subsequently blocked with 1% bovine serum albumin (BSA, Invitrogen) for 2 h. The blocked plates were then incubated for 3 h with recombinant Jagged1-Fc chimera (R & D Systems, Minneapolis, MN) at a concentration of 1 μg/mL. An equal amount of IgG-Fc fragment (Jackson ImmunoResearch, West Grove, PA) was incubated on the control plates. After washing 3 times with PBS, cells were immediately seeded onto the coated plates, and LPS (1 μg/mL) was added on day 4 of cell culture.

In both experiments, 24 h after LPS stimulation, RNA was extracted using RNeasy kit from cells in part of the wells for qPCR analysis of *Ccl2* mRNA expression, while supernatants were collected for CCL2 protein measurement using Quantikine Mouse CCL2/JE/MCP-1 Immunoassay kit (R&D Systems Inc, Minneapolis, MN), normalized to total protein amount in the supernatants (*N* = 4–9 independent cultures per group). For the rest of the wells, coverslips were collected 24 h after LPS stimulation for detecting NICD expression (*N* = 5–9 culture wells per group). Cells were fixed with 100% ice-cold methanol for 10 min, followed by immunofluorescence (IF) staining for NICD.

### Intra-articular (IA) injection of LPS

Under isoflurane anesthesia, LPS (3 μg in 3 μL saline) was injected IA into the right knees of 12-week old naïve male mice, as described [22], and control mice were injected with saline only (*N* = 6 mice per group). Knee hyperalgesia was tested in a 24-h time-course after injection. Three additional independent batches of mice also received LPS or saline control injected IA. RNA was extracted from the ipsilateral L3-L5 DRG 4 h after LPS injection for qPCR analysis of *Ccl2* and Notch signaling genes (batch 1, *N* = 6 mice per group). Proteins were extracted from the L3-L5 DRG 6 and 24 h after LPS injection for ELISA detection of CCL2 (batch 2, *N* = 4 or 5 mice per group), or 24 h after LPS injection for ELISA detection of NICD and CCL2 in the tissue lysates (batch 3, *N* = 4 mice per group).

To detect the effect of inhibition of Notch signaling on LPS-induced changes, DAPT (Catalog# S2215, Selleck Chemical, Houston, TX, 100 μg in 3 μL DMSO) was injected into the right knees of mice one hour prior to LPS injection, while the control group received 3 μL vehicle (*N* = 8 mice per group). Knee hyperalgesia was tested in a 24-h time-course after injection. Proteins were extracted from the ipsilateral DRG 24 h after LPS injection for measuring the levels of CCL2 and NICD in the DRG (*N* = 5 mice per group).

### Assessment of knee hyperalgesia

Knee hyperalgesia was measured using a Pressure Application Measurement (PAM) device (Ugo Basile, Varese, Italy), as previously described [22]. Briefly, mice were restrained by hand and the hind paw was lightly pinned with a finger in order to hold the knee in flexion at a similar angle for each mouse. With the knee in flexion, the PAM transducer was pressed against the medial side of the ipsilateral knee while the operator’s thumb lightly held the lateral side of the knee. The PAM software guided the user to apply an increasing amount of force at a constant rate (30 g/s), up to a maximum of 450 g. If the mouse tried to withdraw its knee, the force at which this occurred was recorded. If the mouse did not try to withdraw, the maximum possible force of 450 g was assigned. Two measurements were taken per knee and the withdrawal force data were averaged. The operators (S.I. and J.L.) were blinded to the treatment groups.

### Notch signaling gene expression in DRG

Ipsilateral L3-L5 DRG were collected 12 or 26 weeks after DMM or sham surgery (*N* = 6 mice per group at 12 weeks after surgery, and *N* = 3 mice per group at 26 weeks after surgery). Genes in the canonical Notch signaling pathway that were identified in the microarray study to be differentially expressed in DRG after DMM were selected for quantitative reverse transcription polymerase chain reaction (qRT-PCR), including *Jag1, Adam17, Rbpj* and *Hes1* [7]. Ipsilateral L3-L5 DRG from each mouse were pooled and homogenized in Trizol (Invitrogen, Carlsbad, CA) on ice, vigorously mixed with chloroform (Sigma, St. Louis, MO), and centrifuged for 15 min at 12,000x*g* at 4 °C. RNA was extracted from the upper aqueous phase using RNeasy kit (Qiagen, Hilden, Germany). The ratios of A260/A280, which were assessed for the quality of RNA, ranged from 1.86 to 2.03, with an average of 1.95. Reverse transcription was performed using RT^2^ first strand kit (Qiagen). Quantification of mRNA was conducted on a Bio-Rad CFX96 machine using the Qiagen SYBR Green qPCR master mix and the RT^2^ primer assays (Supp. Tab. 1). *Gapdh* was used as an internal control for normalization of target gene expression. The comparative 2^−ΔCT^ method was utilized for relative quantitation of gene levels of expression, presented as 2^−ΔCT(Gene of interest – *Gapdh*)^.

To localize the expression of *Jag1, Notch1* and *Hes1*, RNA in situ hybridization (ISH) was performed in 4% paraformaldehyde (PFA)-fixed L4 DRG frozen sections from mice 26 weeks after DMM or sham surgery (*N* = 3 mice per group) using RNAscope™ Multiplex Fluorescent v2 kit and RNAscope™ probes (Advanced Cell Diagnostics, Newark, CA), according to the manufacturer’s instructions. Hybridization signals were detected using Opal fluorophores (Akoya Biosciences, Marlborough, MA). Slides were mounted with ProLong Gold Antifade Mountant (Life Technologies, Eugene, OR) and viewed with an Olympus FV10i confocal laser scanning microscope (Tokyo, Japan).

For quantification of mRNA expression in neurons, H-score (ranged from 0 to 400) was calculated as sum of each (bin number x percentage of cell per bin) according to the 0–4 five-bin scoring system recommended by the manufacturer [23]. Results are presented as an average of the findings from two of the authors (J.L. and L.W.), who were blinded to sample information.

### Active Notch protein, NICD, in DRG

Protein levels of NICD, the active form of Notch protein, in L3-L5 DRG from mice 12 or 26 weeks after DMM or sham surgery (*N* = 4–7 mice per group) were assessed using PathScan Cleaved Notch1 Sandwich ELISA Kit (Cell Signaling Technology, Danvers, MA) following the manufacturer's instructions. Proteins were extracted from DRG using 1 × Cell Lysis Buffer (Catalog# 9803, Cell Signaling Technology). NICD levels in DRG cell lysates are presented as absorbance at 450 nm normalized to total protein, which was measured using the BCA (bicinchoninic acid) assay (Thermo Fisher, Rockford, IL).

To detect NICD in DRG, IF analysis was performed on L4 DRG from mice 12 or 26 weeks after DMM or sham surgery (*N* = 3 mice per group). Mice were perfused transcardially with PBS followed by 4% PFA. The spinal column was dissected and postfixed in 4% PFA overnight followed by cryopreservation in 30% sucrose in PBS. L4 DRGs were embedded with OCT compound (Fisher Healthcare), frozen with dry ice, and cut into 12-μm sections. Sections were stained overnight at 4 °C with an antibody against active Notch (NICD) (Abcam Cat# ab52627, RRID:AB_881725; 1:200), followed by an Alexa Fluor 633-conjugated secondary antibody (Invitrogen, 1:1000) for 1 h at room temperature. The slides were mounted with Vectashield mounting medium with DAPI (Vector Laboratories, Burlingame, CA). Fluorescent signals were quantified as mean fluorescence intensity (MFI) using ImageJ [24]. For each DRG, 2 or 3 sections were evaluated and averaged.

### IA injection of DAPT in DMM joints

To detect the effect of inhibition of Notch signaling on DMM-induced OA pain, DAPT (100 μg in 5 μL DMSO), was injected IA into the right knees, 12 weeks after DMM. DAPT was administered IA, aiming to investigate the DRG that directly innervate the knee joints. IA injection also limits the systemic effects of Notch signaling inhibition and other γ-secretase substrates inhibition by DAPT. Knee hyperalgesia was tested in a 24-h time-course after injection (*N* = 6 mice per group).

In another experiment, mice were injected IA with DAPT 12 weeks after DMM or sham surgery. RNA was extracted from the ipsilateral L3-L5 DRG 6 h after injection for *Ccl2* qPCR analysis (*N* = 6 mice per group), or proteins were extracted from the L3-L5 DRG 24 h after injection for CCL2 and NICD ELISA analysis (*N* = 7 or 5 mice per group).

### Statistical analysis

Results are presented as mean ± SEM. Statistical analyses were performed to compare two groups using unpaired two-tailed Student's *t*-tests at a significance level of *p* < 0.05. Time-course knee hyperalgesia measurements were analyzed by two-way repeated measures ANOVA with Šidák *post hoc* test at a significance level of *p* < 0.05, and area under the curve (AUC) over the time course was analyzed using unpaired two-tailed Student's *t*-tests.

## Results

### LPS stimulation increases NICD in DRG cultures, and induces CCL2 expression in a partly Notch-dependent manner

We have previously reported that TLR4 stimulation with LPS results in increased production of the pro-algesic chemokine, CCL2, in cultured DRG cells [17]. We used the same approach to start exploring the potential link between Notch signaling and TLR activation in DRG cells by testing whether LPS activates Notch signaling in cultured DRG cells, and whether LPS-induced CCL2 expression is dependent on activation of Notch signaling.

**Fig. 1:**
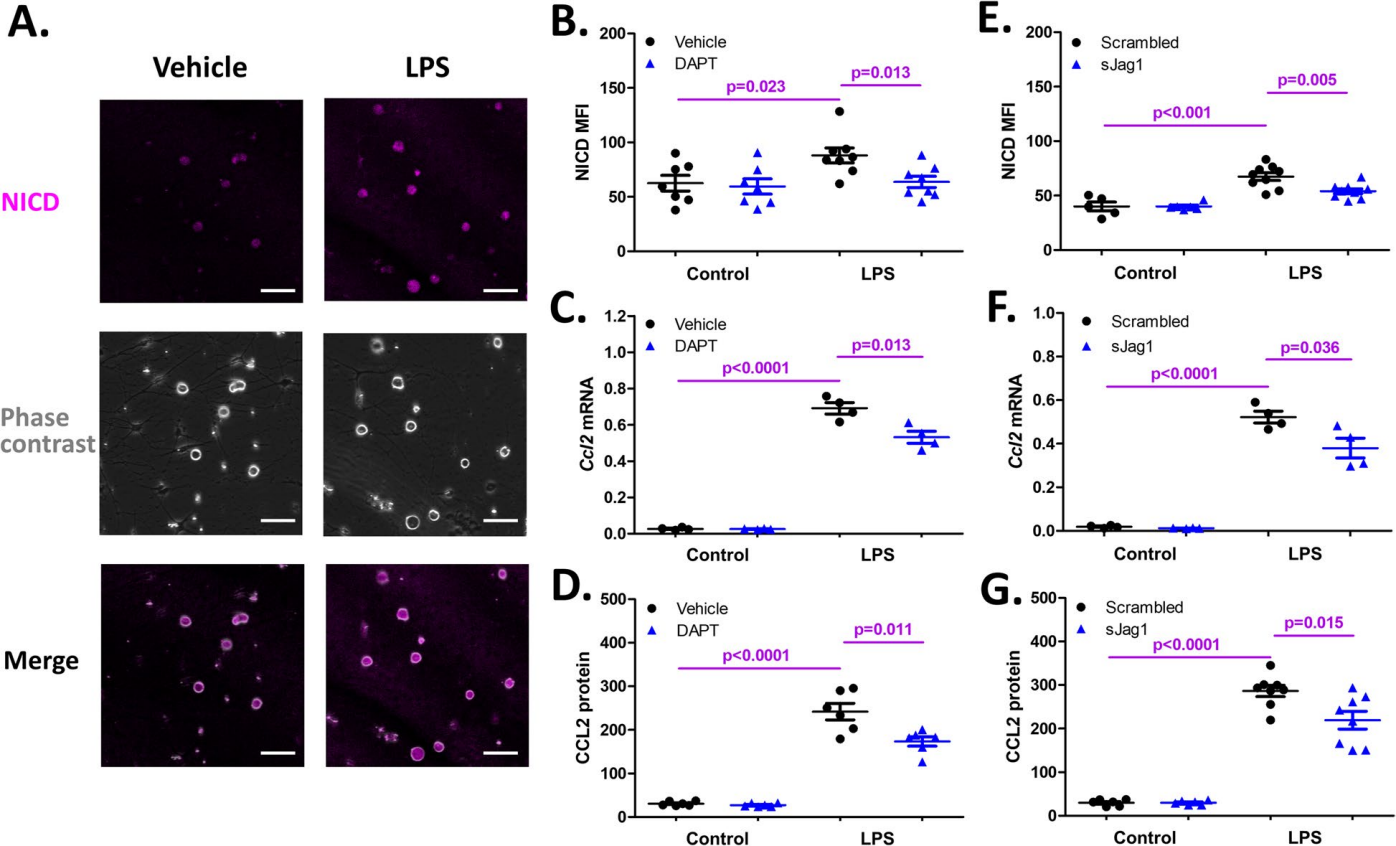
Effects of inhibition of Notch signaling on TLR4-mediated increases of CCL2 in DRG cell cultures. **A** NICD IF staining of the DRG cells treated with LPS (1 μg/mL) for 24 h. Scale bars, 50 μm. **B-D** Effects of inhibiting Notch signaling by DAPT (25 μM) on LPS-induced NICD (quantified as MFI and normalized to the control without LPS and DAPT treatment) (**B**), *Ccl2* mRNA (**C**), and CCL2 protein (**D**) in cultured DRG cells (*N* = 4–8 cultures per treatment group). **E–G** Effects of inhibiting Notch signaling by sJag1 (40 μM) on LPS-increased NICD MFI (**E**), *Ccl2* mRNA (**F**), and CCL2 protein (**G**) (*N* = 4–9 cultures per group)

First, L3-L5 DRG cells from naïve 10-week old male C57BL/6 mice were isolated, cultured, and stimulated with LPS (1 μg/mL) for 24 h. This led to increased NICD expression in DRG cells (representative images in Fig. 1A), with a higher MFI (88.07 ± 6.59) compared to the controls (62.61 ± 7.17) (*p* = 0.023, *N* = 7 or 8 cultures per treatment group) (Fig. 1B). Consistent with previous findings [17], LPS stimulation increased *Ccl2* mRNA expression in these cell cultures, as well as release of CCL2 protein into the culture supernatants, compared to controls treated with vehicle (Fig. 1C-D).

Next, to test the effect of inhibition of Notch signaling on TLR4 stimulation, we blocked Notch signaling with the γ-secretase inhibitor, DAPT, or with soluble Jagged1. Pretreatment of the cells with DAPT (25 μM) attenuated the LPS-induced increase of NICD, *Ccl2* mRNA, and CCL2 protein (*p* = 0.013, *p* = 0.013, and *p* = 0.011, respectively, *N* = 4–8 independent cultures per treatment group) (Fig. 1B-D). Similarly, blocking Notch ligand-receptor interaction with soluble Jagged1 (40 μM) also suppressed LPS-induced increases of NICD, *Ccl2* mRNA, and CCL2 protein when compared to scrambled peptide control (*p* = 0.005, *p* = 0.036, and *p* = 0.015, respectively, *N* = 4–9 cultures per group) (Fig. 1E-G).

**Fig. 2:**
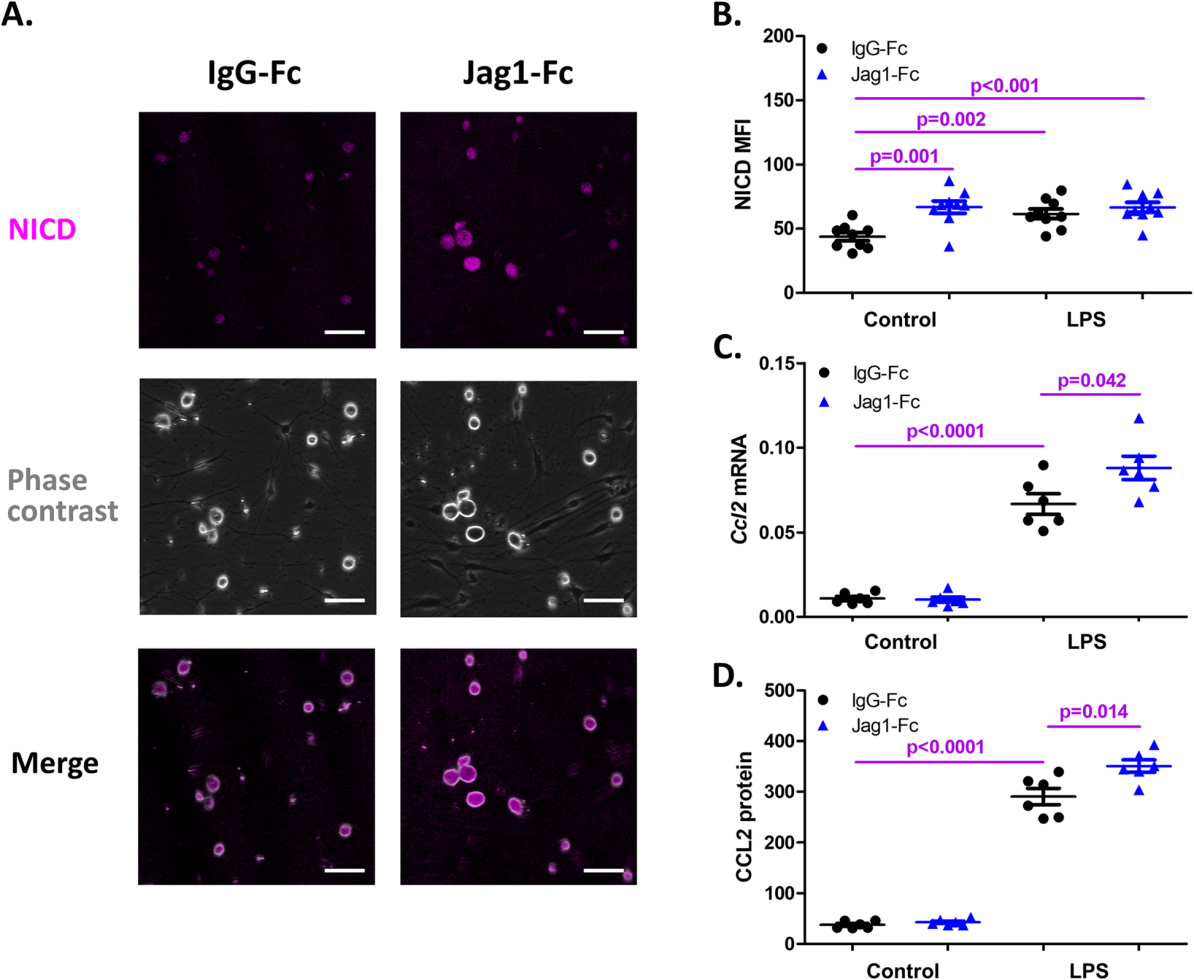
Effects of activation of Notch signaling on TLR4-mediated increases of CCL2 in DRG cell cultures. **A** NICD IF staining of DRG cells cultured with immobilized Jag1-Fc for 4 days. Representative images from each culture group are shown (*N* = 9 cultures per group). Scale bars, 50 μm. **B-D** Effects of activating Notch signaling with immobilized Jag1-Fc on LPS-induced NICD MFI (**B**), *Ccl2* mRNA (**C**), and CCL2 protein (**D**) (*N* = 6–9 per group). The controls were immobilized with IgG-Fc

Conversely, we used immobilized Jagged1 to activate Notch signaling in DRG cultures. The activation was confirmed by the increase of NICD, as shown in representative cultures in Fig. 2A and quantified in Fig. 2B (MFI 61.62 ± 3.50 in Jagged1 activated cultured, compared to MFI 43.77 ± 3.07 in cells incubated with immobilized IgG-Fc (*p* = 0.001, *N* = 9 cultures per group). Immobilized Jagged1 enhanced LPS-induced *Ccl2* mRNA expression and CCL2 protein, when compared to cells incubated with immobilized IgG-Fc (*p* = 0.042 and *p* = 0.014, respectively, *N* = 6 cultures per group) (Fig. 2C-D).

Taken together, these in vitro findings suggest that LPS stimulation activates the Notch signaling pathway in cultured DRG cells, and Notch signaling is involved in CCL2 synthesis induced by LPS.

### IA injection of LPS induces knee hyperalgesia and activates Notch signaling in DRG

**Fig. 3:**
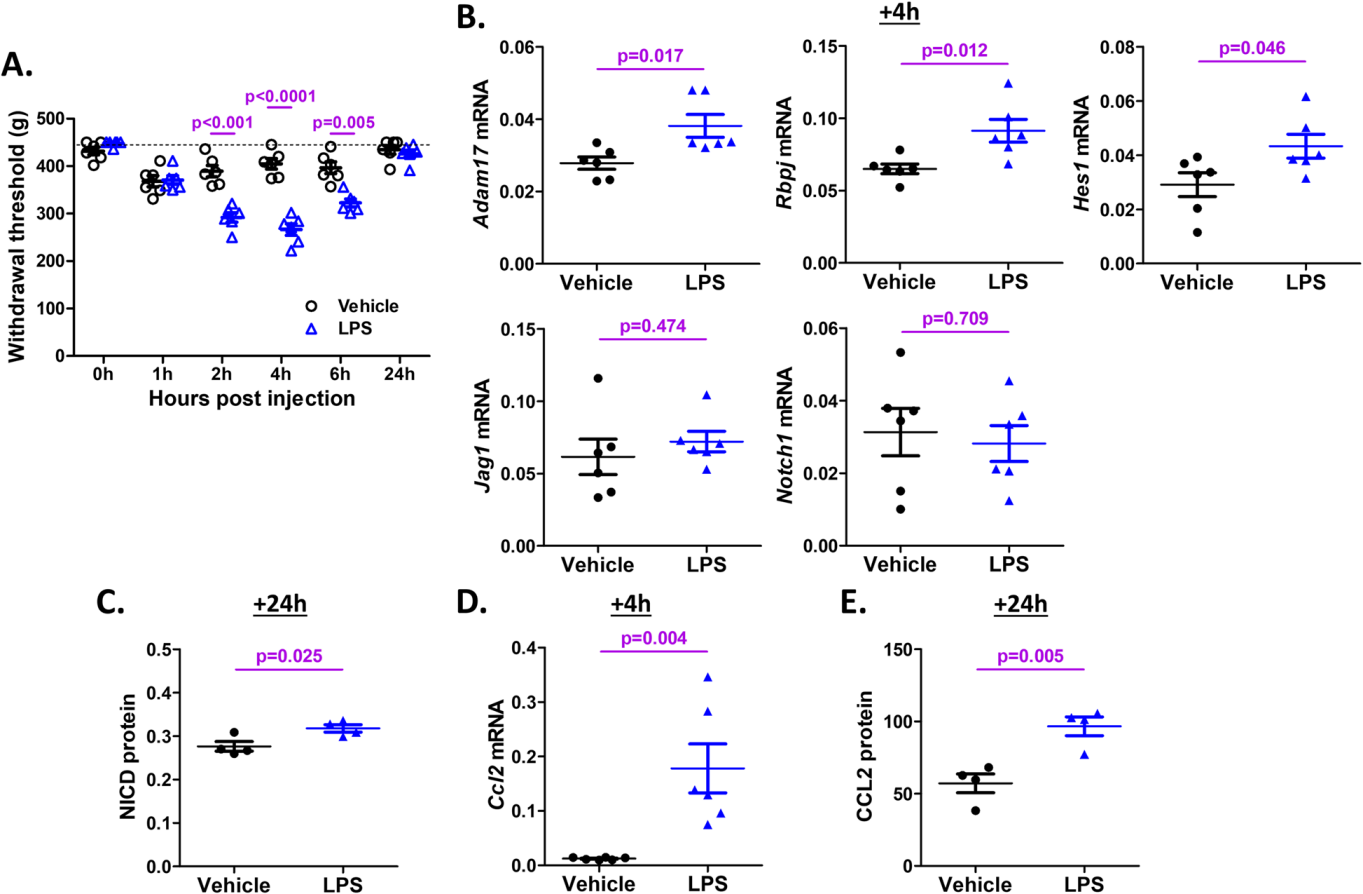
Intra-articular injection of LPS activates Notch signaling in knee-innervating DRG of naïve mice. **A** Knee hyperalgesia 24-h time-course after IA injection of LPS (3 μg) or vehicle in 10-week old naïve mice (*N* = 6 mice per group). 450 g is the threshold baseline for naïve mice (dashed line). **B** Gene expression of *Adam17*, *Rbpj*, *Hes1*, *Jag1*, and *Notch1* in the ipsilateral L3-L5 DRGs 4 h after IA injection of LPS or vehicle (*N* = 6 mice per group). **C** Protein levels of NICD in the pooled L3-L5 DRG tissue lysates 24 h after IA injection of LPS or vehicle (*N* = 4 mice per group). NICD levels are presented as absorbance value at 450 nm normalized to total protein (A450/mg total protein). **D** Gene expression of *Ccl2* in DRG 4 h after IA injection of LPS or vehicle (*N* = 6 mice per group). **E** Protein levels of CCL2 in DRG tissue lysates 24 h after IA injection of LPS or vehicle (*N* = 4 mice per group). CCL2 levels are presented as CCL2 protein normalized to total protein (pg CCL2/mg total protein)

In order to assess whether the in vitro findings in DRG cultures can be translated in vivo, we injected LPS intra-articularly into the knee joint cavity of naïve mice, and monitored the effect on Notch signaling in knee-innervating DRGs, as well as on knee hyperalgesia. LPS (3 μg in 3 μl saline) injection into the right knee joint of 12-week old naïve male C57BL/6 mice caused transient knee hyperalgesia, which peaked after 4 h and returned to baseline 24 h after injection (Fig. 3A) (two-way ANOVA test: *p* < 0.05 *vs.* saline control; *post hoc* test: *p* < 0.001, *p* < 0.0001, and *p* = 0.005 at 2, 4, and 6 h after injection, respectively. *N* = 6 mice per group).

We then determined whether upregulation of Notch signaling genes in the DRG was observed in this model of transient knee joint pain. To this end, LPS or vehicle (*N* = 10 mice per group) was injected IA, and ipsilateral L3-L5 DRG were collected and pooled 4 h later for mRNA analysis (*N* = 6 mice per group), or 24 h after injection for protein analysis in DRG lysates (*N* = 4 mice per group). We found increased mRNA expression of *Adam17*, *Rbpj*, and *Hes1* in DRG 4 h after LPS administration compared to saline (*p* = 0.017, 0.012, and 0.046, respectively), while *Jag1* and *Notch1* genes were unchanged (Fig. 3B). Quantification of NICD protein levels by ELISA in the lysates revealed that NICD levels were increased in LPS-injected mice compared to vehicle controls (*p* = 0.025) (Fig. 3C).

We have previously shown that CCL2 is upregulated in the DRG that innervate painful knees, in the DMM model [18] as well as in knee hyperalgesia induced by TLR2 stimulation [22]. Hence, we measured CCL2 expression in the L3-L5 DRG after intra-articular LPS injection. We found that LPS-induced hyperalgesia was accompanied by increased CCL2 expression in the DRG: *Ccl2* mRNA was upregulated 4 h after LPS injection, the time point when knee hyperalgesia peaked (Fig. 3D), and CCL2 protein levels were increased both 6 and 24 h after injection (Supp. Figure 1 and Fig. 3E).

These results show that IA injection of LPS models transient knee pain, and this is associated with activation of the Notch signaling pathway. Furthermore, knee pain and Notch signaling are accompanied by upregulation of the pro-algesic chemokine, CCL2, in knee-innervating DRG.

### Local administration of DAPT reduces LPS-induced knee hyperalgesia and CCL2 expression in DRG

**Fig. 4:**
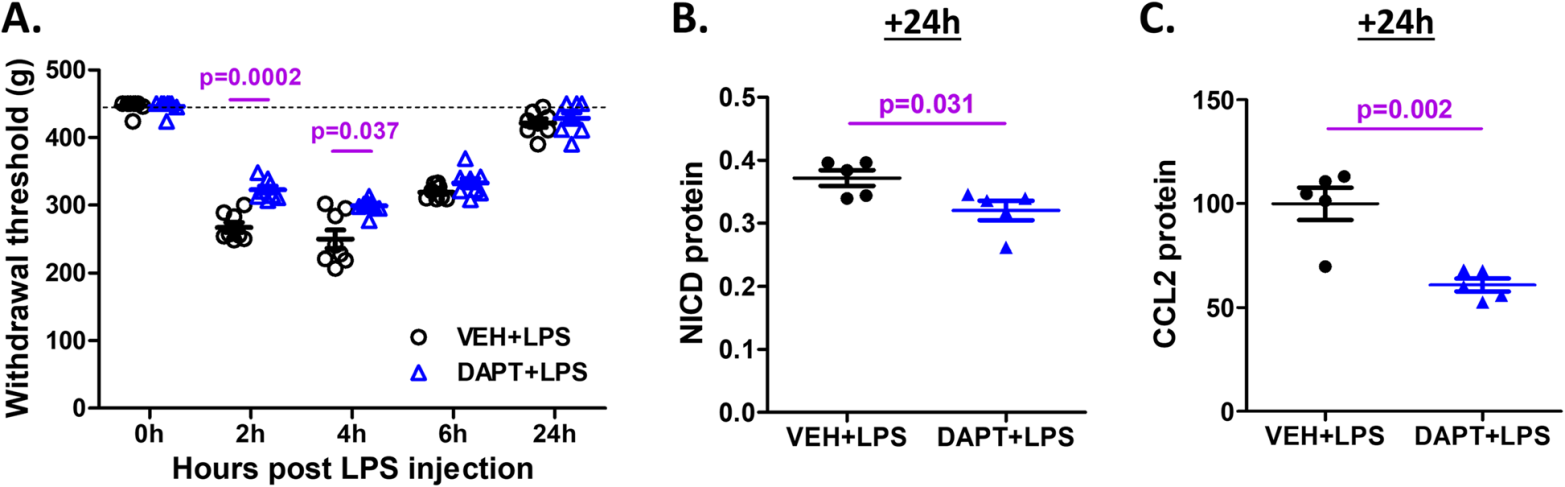
Effects of inhibition of Notch signaling on LPS-induced knee hyperalgesia and CCL2 expression in DRG. Mice were pre-injected IA with DAPT (100 μg) or vehicle one hour prior to LPS injection (3 μg). **A** Time-course of knee hyperalgesia following IA injection of DAPT and LPS (*N* = 8 mice per group). **B, C** Protein levels of NICD (**B**) and CCL2 (**C**) in the DRG tissue lysates 24 h after LPS injection (*N* = 5 mice per group)

Since IA administration of LPS in the knee was associated with knee hyperalgesia and Notch signaling activation in the lumbar DRG, we evaluated whether blocking Notch signaling through DAPT had an effect on LPS-induced knee hyperalgesia. We injected DAPT (100 μg) into the knees one hour prior to LPS administration, and monitored knee hyperalgesia over a 24-h time-course, as before. We found that pretreatment with DAPT alleviated LPS-induced hyperalgesia, with significant reductions 2 and 4 h after LPS injection (*p* = 0.0002 and 0.037, respectively, *N* = 8 mice per group) (Fig. 4A). In order to confirm that this effect was mediated through inhibition of the Notch signaling pathway, we showed that pre-injection of DAPT also decreased the protein levels of NICD compared to vehicle control (*p* = 0.031, *N* = 5 mice per group) (Fig. 4B), and this was accompanied by decreased CCL2 protein in the ipsilateral DRG collected 24 h after LPS injection, compared to vehicle (*p* = 0.002, *N* = 5 mice per group) (Fig. 4C).

### Notch signaling in lumbar DRG after DMM, and its role in knee hyperalgesia

The results described above suggest that the activation of the Notch signaling pathway in knee-innervating DRG has a functional role in knee pain through TLR activation and subsequent production of the pro-algesic chemokine, CCL2. Therefore, we aimed to broaden these findings to a model of experimental OA, induced by DMM surgery.

#### Expression of genes in the Notch signaling pathway in the DRG after DMM *vs.* sham surgery

**Fig. 5:**
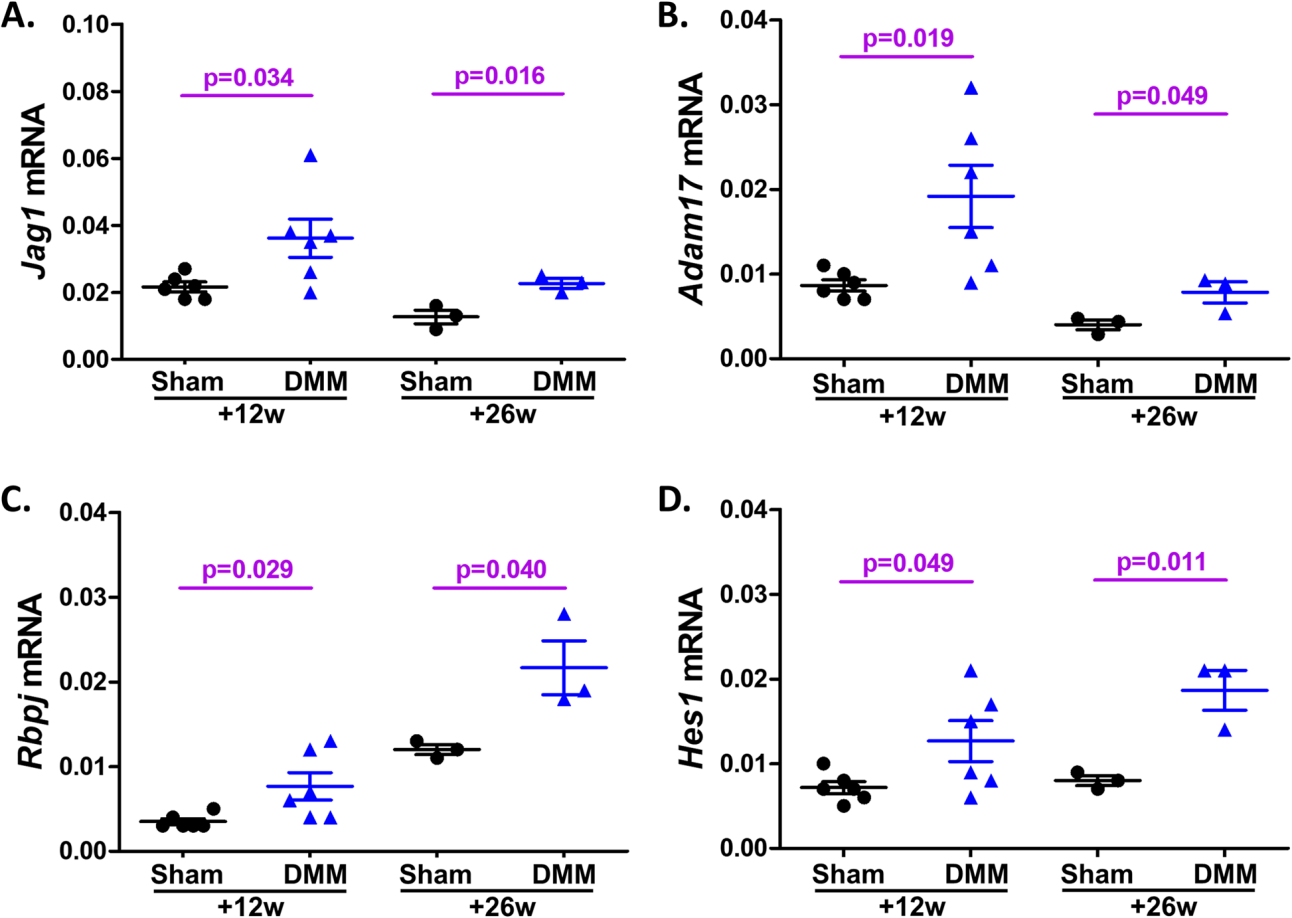
Notch signaling pathway genes are upregulated in knee-innervating DRG after DMM surgery. Quantitative RT-PCR analysis of *Jag1* (**A**), *Adam17* (**B**), *Rbpj* (**C**), and *Hes1* (**D**) mRNA expression in ipsilateral L3-L5 DRGs harvested from mice 12 and 26 weeks after DMM or sham surgery (*N* = 6 mice per group for 12 weeks, and *N* = 3 mice per group for 26 weeks after surgeries). Gene expression is presented as 2^−ΔCT(Gene of interest – *Gapdh*)^. Each dot represents one mouse

First, we explored whether genes in the canonical Notch signaling pathway are upregulated in a surgical model of OA induced by DMM—as previously suggested by our microarray studies, which revealed that genes in the canonical Notch signaling pathway are differentially regulated in the DRG from mice with late-stage OA after DMM compared to sham-operated controls [7], including the Notch ligand, *Jag1*, the S2 Notch cleavage enzyme, *Adam17*, the Notch transcription moderator, *Rbpj*, and the Notch target gene, *Hes1*. Here, we sought to validate these findings using qRT-PCR to assess gene expression of these Notch signaling components in the ipsilateral L3-L5 DRG after DMM or sham surgery. Twelve weeks after DMM *vs.* sham surgery, DRG mRNA levels were increased for *Jag1* (*p* = 0.034), *Adam17* (*p* = 0.019), *Rbpj* (*p* = 0.029), and *Hes1* (*p* = 0.049) (Fig. 5A-D). These genes were still upregulated 26 weeks after DMM surgery (*p* = 0.016, *p* = 0.049, *p* = 0.040, and *p* = 0.011, respectively) (Fig. 5A-D), confirming the findings from the microarray study.

**Fig. 6:**
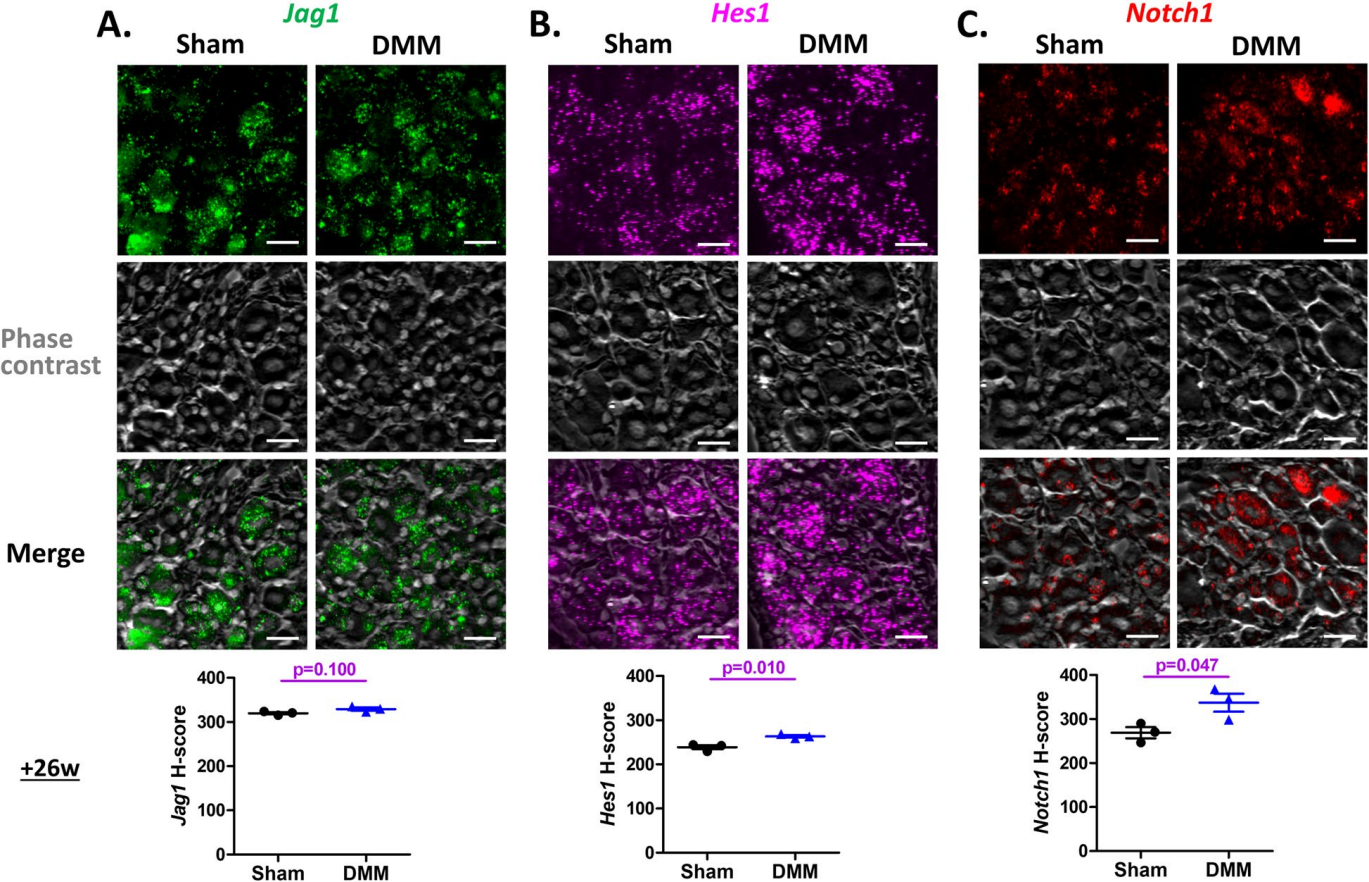
Notch signaling genes are expressed in DRG neurons after DMM surgery. RNA in situ hybridization analysis of *Jag1* (**A**), *Hes1* (**B**), and *Notch1* (**C**) in the L4-DRG of mice 26 weeks after DMM or sham surgery. Representative images of each group are shown (*N* = 3 mice per group). Scale bars, 25 μm. RNA expression in the neurons was quantified using H-score

We then used RNAscope to detect in situ expression of Notch signaling components (*Jag1*, *Hes1*, and *Notch1*) in the ipsilateral L4 DRG, 26 weeks after surgery. Neurons were identified by DAPI staining and phase contrast images, which is comparable to the method of identification of neurons with IF staining of a neuronal marker, PGP9.5 (Supp. Figure 2). *Jag1* was expressed in DRG neurons both after sham and DMM surgery, and the H-scores revealed no difference in expression level (319.96 ± 2.50 after sham *vs.* 329.25 ± 3.58 after DMM) (Fig. 6A). *Hes1* was also detected in DRG neurons, and increased 26 weeks after DMM compared to sham surgery, with H-scores of 238.9 ± 4.6 after sham *vs.* 263.5 ± 2.9 after DMM (*p* = 0.010) (Fig. 6B). Finally, RNAscope detected neuronal *Notch1* expression, which increased 26 weeks after DMM, with an H-score of 337.3 ± 20.5 *vs.* 268.9 ± 12.6 after sham surgery (*p* = 0.047) (Fig. 6C).

Since RNAscope ISH suggested neuronal expression of these Notch signaling components, we examined neuronal expression of additional Notch pathway genes in a recently completed single cell RNA sequencing (scRNAseq) analysis of L3-L5 DRGs of male naïve mice at 18 weeks of age [25]. The results showed that Notch ligand *Dll3*, Notch transcriptional binding complex genes (*Rbpj*, *Maml3*), target gene (*Hey1*), and γ-secretase complex component genes (*Ncstn*, *Aph1a*, *Psen1*, *Psen2*) are predominantly expressed in nociceptors (Supp. Figure 3A-B). qRT-PCR confirmed the upregulation of many of these genes (*Dll3*, *Rbpj*, *Maml3*, and *Aph1a*) in DRG 26 weeks after DMM (Supp. Figure 3C-J).

#### Active Notch protein is increased in DRG neurons after DMM

**Fig. 7:**
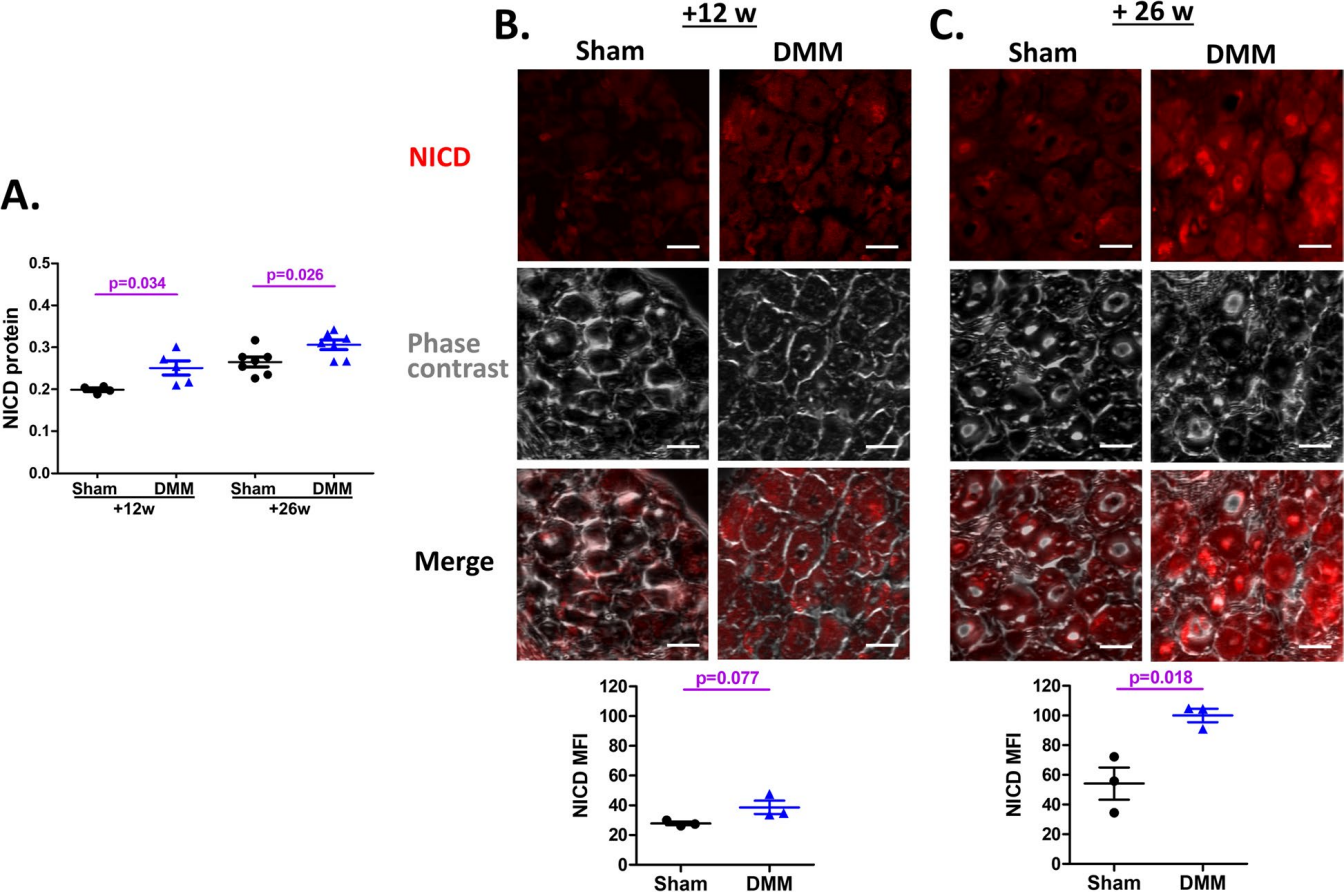
Activated Notch protein (NICD) is increased in DRGs of mice after DMM surgery. **A** ELISA assessment of NICD in tissue lysates of pooled L3-L5 DRGs from mice 12 and 26 weeks after DMM or sham surgery (*N* = 4–7 mice per group). NICD levels are presented as absorbance at 450 nm normalized to total proteins. **B**, **C** NICD immunofluorescence staining in the L4-DRG from mice 12 weeks (**B**) and 26 weeks (**C**) after DMM or sham surgery. Representative images of each group are shown (*N* = 3 mice per group). Scale bars, 25 μm. Fluorescence signals were quantified as mean fluorescence intensity (MFI) (each dot represents one mouse)

Having established that the Notch signaling pathway is regulated in DRG neurons after DMM surgery, we sought to assess whether the upregulation of these genes is accompanied by the release of the Notch intracellular domain, NICD, which is generated when γ-secretase cleaves the Notch receptor [10]. Here, we confirmed the presence of NICD by measuring NICD protein levels by ELISA in the tissue lysates of ipsilateral L3-L5 DRG. We found an increase 12 and 26 weeks after DMM, compared to sham-operated mice (12 weeks: *p* = 0.034, *N* = 4 sham and 5 DMM; 26 weeks: *p* = 0.026, *N* = 7 mice per group) (Fig. 7A).

To confirm that NICD is generated after DMM, we performed an independent experiment, assessing IF staining on cryo-sections of the ipsilateral L4 DRG, 12 and 26 weeks after surgery. Increased NICD staining was shown in the neuronal cell bodies after DMM compared to sham-operated controls, with an MFI of 27.87 ± 1.09 after sham *vs.* 38.66 ± 4.43 after DMM 12 weeks after surgery (*p* = 0.077, *N* = 3 mice per group) (Fig. 7B), and an MFI of 54.17 ± 10.89 26 weeks after sham surgery *vs.* 100.08 ± 4.51 after DMM (*p* = 0.018, *N* = 3 mice per group) (Fig. 7C).

#### IA administration of DAPT reduces knee hyperalgesia after DMM surgery, and suppresses CCL2 expression in the DRG

**Fig. 8:**
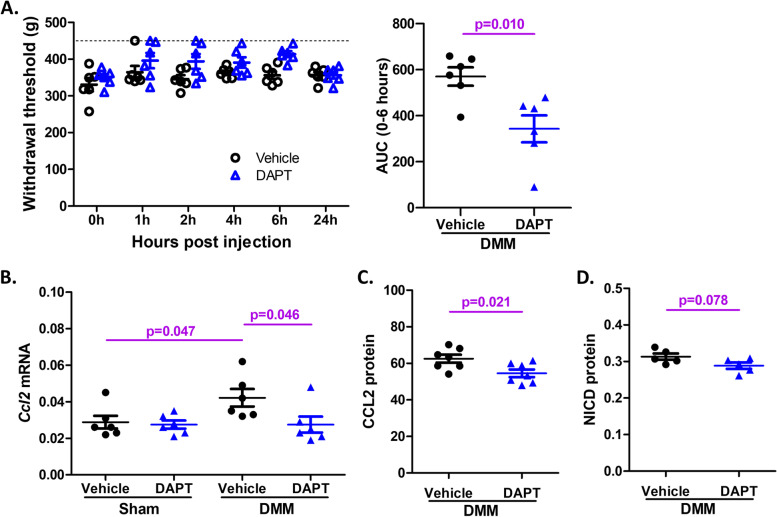
Intra-articular injection of DAPT reduces DMM-induced knee hyperalgesia and attenuates CCL2 expression in ipsilateral DRG. **A** Knee hyperalgesia 24-h time-course tests after IA injection of DAPT (100 μg) or vehicle in mice 12 weeks after DMM surgery (*N* = 6 mice per group). 450 g is the threshold baseline for naïve mice (dashed line). Area under the curve (AUC) was calculated for 0–6 h to assess knee hyperalgesia. **B** *Ccl2* mRNA expression in the ipsilateral L3-L5 DRG from mice 12 weeks post-surgery and 6 h after IA injection of DAPT or vehicle (*N* = 6 mice per group). **C, D** Protein levels of CCL2 (**C**) and NICD (**D**) in the pooled L3-L5 DRG tissue lysates of the DMM mice 24 h after DAPT injection (*N* = 7 or 5 mice per group)

Finally, we evaluated whether blocking Notch signaling in the DMM model through IA injection of DAPT had an effect on knee hyperalgesia. Twelve weeks after DMM, mice showed robust hyperalgesia at the operated knee, manifest as a lower withdrawal threshold, as we have described before [26] (Fig. 8A). At that time point, we assessed the effect of IA injection of DAPT in a 24-h time course. Compared to vehicle, IA administered DAPT (100 μg) attenuated knee hyperalgesia, as indicated by a higher withdrawal threshold (two-way ANOVA test, *p* = 0.336 *vs.* vehicle; AUC for 0 to 6 h, *t*-test, *p* = 0.010 *vs.* vehicle. *N* = 6 mice per group) (Fig. 8A). Knee hyperalgesia returned to pre-injection levels by 24 h after injection of DAPT (Fig. 8A).

Ipsilateral L3-L5 DRG were collected from another group of mice with the same treatment 6 h after injection for qRT-PCR. *Ccl2* mRNA expression was increased in DMM mice compared to sham group after injection with vehicle (*p* = 0.047, *N* = 6 mice per group) (Fig. 8B), and these increases were attenuated when the DMM-operated knees were injected with DAPT (*p* = 0.046, *N* = 6 mice per group) (Fig. 8B). L3-L5 DRG were also collected and lysed from mice 24 h after DAPT injection for CCL2 and NICD detection in the lysates using ELISA. Compared to vehicle, DAPT injection had a trend to decrease both CCL2 protein (*p* = 0.021, *N* = 7 mice per group) and NICD protein (*p* = 0.078, *N* = 5 mice per group) in DRG (Fig. 8C-D).

## Discussion

Our studies revealed that the TLR4 agonist LPS increased activated Notch protein in cultured DRG neurons from naïve mice. Activation of Notch signaling also occurred following LPS treatment in vivo*,* together with increased expression of the chemokine CCL2, which we have demonstrated occurs during the establishment of painful OA [18]. In DRG cell cultures, inhibition of Notch signaling led to decreased LPS-induced CCL2 production, and activation of Notch signaling led to increased LPS-induced CCL2 synthesis. Similarly, in vivo*,* pre-injection of the γ-secretase inhibitor DAPT, resulted in attenuated LPS-induced knee hyperalgesia and decreased LPS-induced NICD and CCL2 in the lumbar DRG. In the DMM model of OA, qRT-PCR detected increased expression of several genes in the Notch signaling pathway in the L3-L5 DRG—including *Jag1, Adam17, Rbpj,* and *Hes1*—confirming our published microarray studies [7]. In situ hybridization analysis of the DRG showed that *Notch1* and *Hes1* were highly expressed in the neuronal cell bodies after DMM, and ELISA and IF staining showed that NICD was increased in DRG neurons. IA administration of DAPT resulted in inhibition of Notch activation in the knee-innervating DRG accompanied by decreased CCL2 production and a transient reduction of knee hyperalgesia after DMM. Taken together, these findings suggest a role for activation of the Notch signaling pathway in knee joint pain associated with experimental OA. Our results also indicate that Notch signaling may interact synergistically with TLR4 signaling.

Crosstalk between Notch and TLR signaling pathways has been reported in several studies, most of which were focused on macrophages and monocytes under inflammatory conditions [16]. LPS may activate Notch signaling indirectly through upregulating the expression of Notch ligands [27] and receptors [28, 29], or directly through inducing expression of Notch target genes [30]. Conversely, Notch signaling amplifies TLR-mediated pro-inflammatory responses by increasing NF-*κ*B activity [29] and enhancing expression of pro-inflammatory cytokines [28–30]. In a murine model of atherosclerosis, blockade of Notch signaling reduced atherogenesis, associated with diminished CCL2 expression and suppressed NF-κB activation in atherosclerotic lesions [31].

Notch signaling plays a fundamental role in neurogenesis and neuroplasticity [32]. Activation of Notch signaling is involved in the development of neuropathic pain [13, 14]. Interaction between Notch1 and TLR4 signaling pathways in DRG neurons has been reported in diabetic rats with neuropathic pain, where inhibition of either Notch1 or TLR4 signaling attenuated mechanical allodynia and thermal hyperalgesia [33]. In the current study, we showed that an interaction between Notch and TLR4 signaling also exists in the peripheral nervous system in a mouse model of OA. TLR4 signaling in response to DAMPs generated in the osteoarthritic joint may normally activate Notch signaling in lumbar DRG. Activated Notch signaling in sensory afferents may synergize with TLR4 signaling in promoting synthesis of pro-algesic mediators such as CCL2, and thereby mediate the development of knee hyperalgesia. TLR4 stimulation by LPS clearly activates Notch signaling, as is evident from the observed increase of NICD in DRG, and this can be blocked with DAPT.

Of interest, a recently published comparative transcriptome profile analysis revealed conserved enrichment of genes in human and mouse DRG [34]. After reviewing their database of L2 lumbar DRG from male and female human donors with and without neuropathic pain [34, 35], we found that several Notch signaling pathway genes are expressed in human DRG with a TPM (transcripts per million) over 10. Compared to donors with no pain, the Notch target gene *HES1* showed a 1.86-fold increase in male and a 1.65-fold increase in female donors with pain; Notch ligand *JAG1* a 2.66-fold increase in female donors with pain; and Notch moderator *RBPJ* a 2.25-fold increase in females with pain (Supp. Figure 4). These data further support that Notch signaling activation in DRG may be associated with pain.

Neurons located in L3-L5 lumbar DRG innervate the knee joint, but it is not clear from our experiments whether the neurons with increased NICD are those that innervate the knee or other structures. Furthermore, the DRG not only contain the cell bodies of sensory neurons, but also a wide variety of other cell types such as satellite glial cells and immune cells. Given the fact that Notch signaling normally proceeds through cell-to-cell interactions it is quite possible that its signaling influence in this case may not be cell autonomous. Further studies are required to answer this question.

One limitation of our microarray [7] and qRT-PCR data is that they reflect gene expression by all cells in the DRG. Nevertheless, our in situ hybridization results suggest that certain genes such as *Notch1* may be preferentially enhanced in neurons even though the global expression as seen through qPCR did not change. Furthermore, scRNA-seq is a powerful tool for studying cellular diversity in the DRG and our laboratory and other groups have confirmed gene expression profiles of some Notch signaling molecules by scRNAseq analysis of DRG cells from naïve mice [25, 36]. The results revealed that Notch signaling genes are expressed in peptidergic and non-peptidergic nociceptive neurons, including genes encoding Notch ligands, receptors, γ-secretase complex components, transcriptional binding complex components, and downstream targets.

Taken together, our studies revealed activation of Notch signaling in the DRGs that innervate the knee, and suggest a role for the pathway in mediating knee hyperalgesia in OA. These findings add to the existing literature implicating the Notch signaling pathway in OA pathogenesis. It has been reported that the pathway is highly activated in joint tissues, both human and murine, in the course of post-traumatic OA [37, 38]. While physiological Notch signaling is required for long-term maintenance of articular cartilage, enhanced Notch signaling leads to progressive OA pathology in a mouse surgical model [39]. Conditional inactivation of Notch signaling in mouse chondrocytes caused resistance to OA development, and inhibition of Notch signaling by IA injecting DAPT for 10 weeks prevented OA development in mice [37]. Combining our findings on the role of Notch signaling in mediating hyperalgesia in OA mice, it could be expected that modulating Notch signaling may not only alleviate OA pain, but also ameliorate the progression of OA. Notch signaling may therefore be explored as a target for controling both OA pain and progression of joint damage. Large-scale temporal gene expression profiling revealed that Notch activation by overexpression of NICD in chondrocytes not only decreased expression of chondrogenic markers and increased expression of inflammatory factors, but also increased the expression of the gene encoding the neurotrophin, nerve growth factor (NGF) [39]. Since structural changes in joint innervation, including NGF-induced sprouting, have been reported in OA joints and may be related to joint pain [40–42], upregulation of NGF through Notch activation in chondrocytes may constitute another way in which the Notch pathway contributes to OA pain.

## Conclusions

The presented findings suggest that peripheral neuronal Notch signaling contributes to joint pain in murine experimental OA. A model is emerging whereby TLR4 stimulation in the OA joint activates Notch signaling in lumbar DRG. Synergy between Notch signaling and TLR4 signaling promotes joint pain through increased synthesis of the pro-algesic chemokine, CCL2. The Notch signaling pathway in the peripheral nervous system therefore merits deeper exploration as a target for alleviating OA pain.

## Supplementary Information

**Additional file 1:** **Supp. Tab. 1.** List of PCR primers and RNAscope probes used in the qRT-PCR and RISH. **Supp. Fig. 1.** Protein levels of CCL2 in DRG tissue lysates 6 and 24 h after IA injection of LPS or vehicle (*N *=4 or 5 mice per group). CCL2 levels are presented as CCL2 protein normalized to total protein (pg CCL2/mg total protein). **Supp. Fig. 2**. Identification of DRG neurons using phase contrast micrographs and DAPI staining, compared to the method using a neuronal marker PGP9.5 IF staining. Sections were stained overnight at 4°C with an antibody against PGP9.5 (Sigma-Aldrich Cat# SAB4503057, RRID:AB_10761291; 1:200), followed by an Alexa Fluor 488-conjugated secondary antibody (Invitrogen, 1:1000) for 1 h at room temperature. Scale bars, 50 µm. **Supp. Fig. 3. **DRG scRNAseq analysis reveals some Notch pathway genes are specifically or predominantly expressed in nociceptors in DRG. (A) Dot plots showing expression of Notch receptor genes (*Notch1-4*) and ligand genes (*Jag1,2, Dll1,3,4, Dlk1*). Dll3 is specifically expressed in nociceptors. (B) Dot plots showing expression of Notch transcriptional binding complex genes (*Rbpj, Maml3*), target genes (*Hes1, Hey1*) and γ-secretase complex component genes (*Ncstn, Aph1a, Psen1,2, Psenen*) expressed in DRG cells. *Rbpj, Maml3, Hey1, Ncstn, Aph1a, Psen1* and *Psen2* are predominantly expressed in nociceptors. SCHW, Schwann cells; SATG, satellite glial cells; VLMC-like, vascular leptomeningeal like cells; VEC, vascular endothelial cells; VSMCA, vascular smooth muscle cells arterial; NOCI, nociceptors; LDN, large diameter neurons; IMM, immune cells. (C-J) Quantitative RT-PCR analysis of these nociceptor-predominantly expressed gene expression in DRG harvested from mice 26 weeks after DMM or sham surgery (*N *=3 mice per group). Gene expression was presented as 2^-ΔCT(Gene of interest – *Gapdh*)^. Each dot represents one mouse. **Supp. Fig. 4.** Notch signaling genes (*JAG1, NOTCH1, RBPJ,* and *HES1*) in human DRG. Raw data of TPM (transcripts per million) were obtained from comparative transcriptome profile analysis of L2 lumbar DRG of human donors [34, 35]. Gene expression was compared between donors without pain and those with neuropathic pain using unpaired 2-tailed Student's *t*-test. Totally 15 males (4 “no pain” and 11 “pain”) and 12 females (4 “no pain” and 8 “pain”) were analyzed.

## Data Availability

All data generated or analyzed during this study are included in this published article.

## Abbreviations

ADAM: A disintegrin and metalloprotease
CCL2: Chemokine C–C motif chemokine ligand 2
DAMP: Damage-associated molecular pattern
DAPT: *N*-[*N*-(3,5-Diflurophenylacetate)-l-alanyl]-(S)-phenylglycine t-butyl ester
DMM: Destabilization of the medial meniscus
DRG: Dorsal root ganglia
ELISA: Enzyme-linked immunosorbent assay
HES: Hairy and enhancer of split
IA: Intra-articular
IF: Immunofluorescence
LPS: Lipopolysaccharide
MFI: Mean fluorescence intensity
MMTL: Medial meniscotibial ligament
NGF: Nerve growth factor
NICD: Notch intracellular domain
OA: Osteoarthritis
PAM: Pressure application measurement
PBS: Phosphate buffered saline
PFA: Paraformaldehyde
qRT-PCR: Quantitative reverse transcription polymerase chain reaction
RBPJ: Recombination signal binding protein for immunoglobulin kappa J region
RISH: RNA in situ hybridization
scRNA-Seq: Single cell RNA sequencing
sJag1: Soluble Jagged1
TLR: Toll-like receptor
