# Supplementary material for: Notch signaling is activated in knee-innervating dorsal root ganglia in experimental models of osteoarthritis joint pain
Source: Arthritis Res Ther. 2023 Apr 15;25:63. doi: 10.1186/s13075-023-03039-1 (PMC10105425; doi:10.1186/s13075-023-03039-1)
Supplement: Supplementary file 1 — Additional file 1: Supp. Tab. 1. List of PCR primers and RNAscope probes used in the qRT-PCR and RISH. Supp. Fig. 1. Protein levels of CCL2 in DRG tissue lysates 6 and 24 h after IA injection of LPS or vehicle (N =4 or 5 mice per group). CCL2 levels are presented as CCL2 protein normalized to total protein (pg CCL2/mg total protein). Supp. Fig. 2. Identification of DRG neurons using phase contrast micrographs and DAPI staining, compared to the method using a neuronal marker PGP9.5 IF staining. Sections were stained overnight at 4°C with an antibody against PGP9.5 (Sigma-Aldrich Cat# SAB4503057, RRID:AB_10761291; 1:200), followed by an Alexa Fluor 488-conjugated secondary antibody (Invitrogen, 1:1000) for 1 h at room temperature. Scale bars, 50 µm. Supp. Fig. 3. DRG scRNAseq analysis reveals some Notch pathway genes are specifically or predominantly expressed in nociceptors in DRG. (A) Dot plots showing expression of Notch receptor genes (Notch1-4) and ligand genes (Jag1,2, Dll1,3,4, Dlk1). Dll3 is specifically expressed in nociceptors. (B) Dot plots showing expression of Notch transcriptional binding complex genes (Rbpj, Maml3), target genes (Hes1, Hey1) and γ-secretase complex component genes (Ncstn, Aph1a, Psen1,2, Psenen) expressed in DRG cells. Rbpj, Maml3, Hey1, Ncstn, Aph1a, Psen1 and Psen2 are predominantly expressed in nociceptors. SCHW, Schwann cells; SATG, satellite glial cells; VLMC-like, vascular leptomeningeal like cells; VEC, vascular endothelial cells; VSMCA, vascular smooth muscle cells arterial; NOCI, nociceptors; LDN, large diameter neurons; IMM, immune cells. (C-J) Quantitative RT-PCR analysis of these nociceptor-predominantly expressed gene expression in DRG harvested from mice 26 weeks after DMM or sham surgery (N =3 mice per group). Gene expression was presented as 2-ΔCT(Gene of interest – Gapdh). Each dot represents one mouse. Supp. Fig. 4. Notch signaling genes (JAG1, NOTCH1, RBPJ, and HES1) in human DRG. Raw data of TPM (transcripts per mill [file 13075_2023_3039_MOESM1_ESM.docx]

**Supplementary material**

**Supp. Tab. 1.** List of PCR primers and RNAscope probes used in the qRT-PCR and RISH.

**Supp. Fig. 1.** Protein levels of CCL2 in DRG tissue lysates 6 and 24 hours after IA injection of LPS or vehicle (N=4 or 5 per group). CCL2 levels are presented as CCL2 protein normalized to total protein (pg CCL2/mg total protein).

**
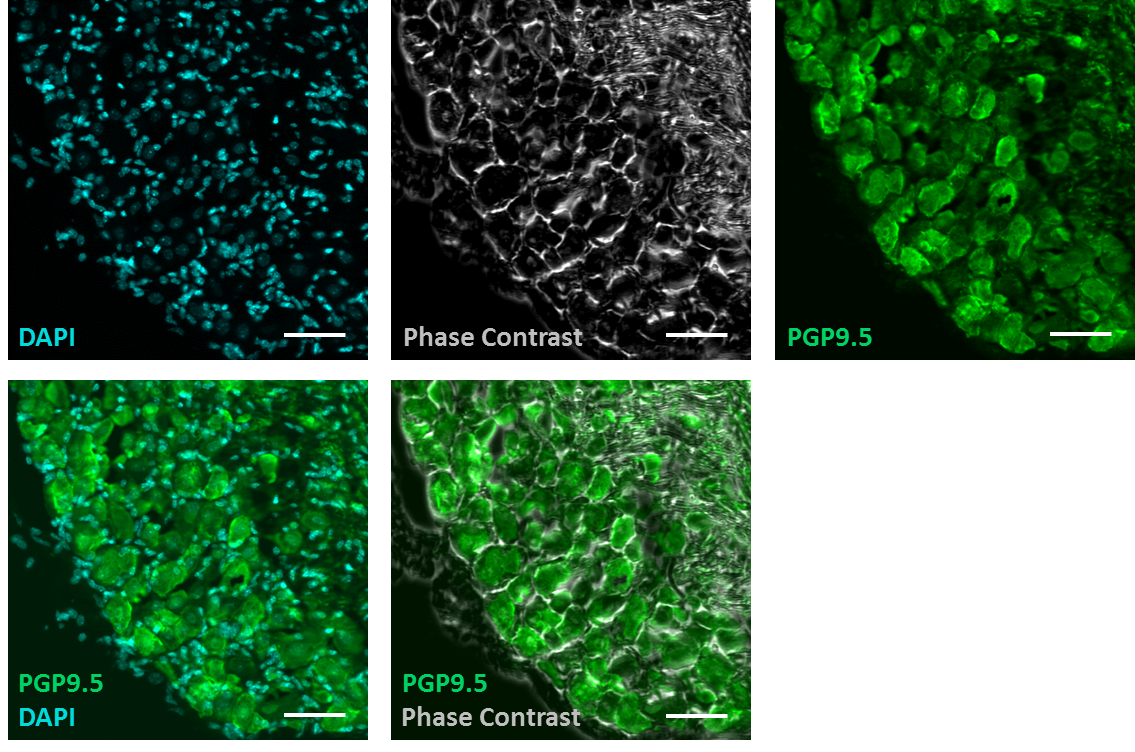
**

**Supp. Fig. 2**. Identification of DRG neurons using phase contrast micrographs and DAPI staining, compared to the method using a neuronal marker PGP9.5 IF staining. Sections were stained overnight at 4°C with an antibody against PGP9.5 (Sigma-Aldrich Cat# SAB4503057, RRID:AB_10761291; 1:200), followed by an Alexa Fluor 488-conjugated secondary antibody (Invitrogen, 1:1000) for 1 hour at room temperature. Scale bars, 50 µm.

**
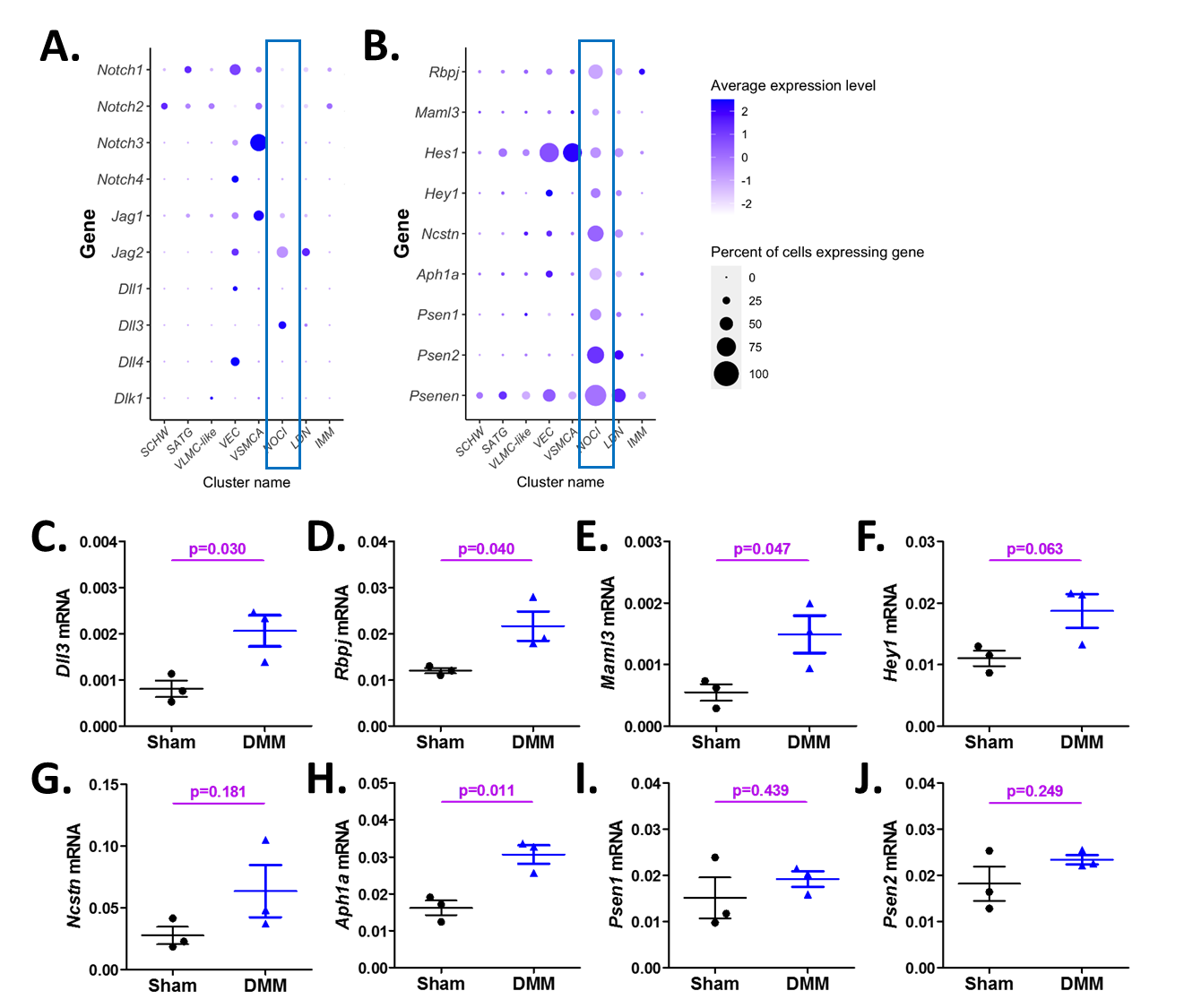
**

**Supp. Fig. 3.** DRG scRNAseq analysis reveals some Notch pathway genes are specifically or predominantly expressed in nociceptors in DRG. (A) Dot plots showing expression of Notch receptor genes (Notch1-4) and ligand genes (Jag1,2, Dll1,3,4, Dlk1). Dll3 is specifically expressed in nociceptors. (B) Dot plots showing expression of Notch transcriptional binding complex genes (Rbpj, Maml3), target genes (Hes1, Hey1) and γ-secretase complex component genes (Ncstn, Aph1a, Psen1,2, Psenen) expressed in DRG cells. Rbpj, Maml3, Hey1, Ncstn, Aph1a, Psen1 and Psen2 are predominantly expressed in nociceptors. SCHW, Schwann cells; SATG, satellite glial cells; VLMC-like, vascular leptomeningeal like cells; VEC, vascular endothelial cells; VSMCA, vascular smooth muscle cells arterial; NOCI, nociceptors; LDN, large diameter neurons; IMM, immune cells. (C-J) Quantitative RT-PCR analysis of these nociceptor-predominantly expressed gene expression in DRG harvested from mice 26 weeks after DMM or sham surgery (N=3 per group). Gene expression was presented as 2^-ΔCT(Gene of interest – Gapdh)^. Each dot represents one mouse.

**
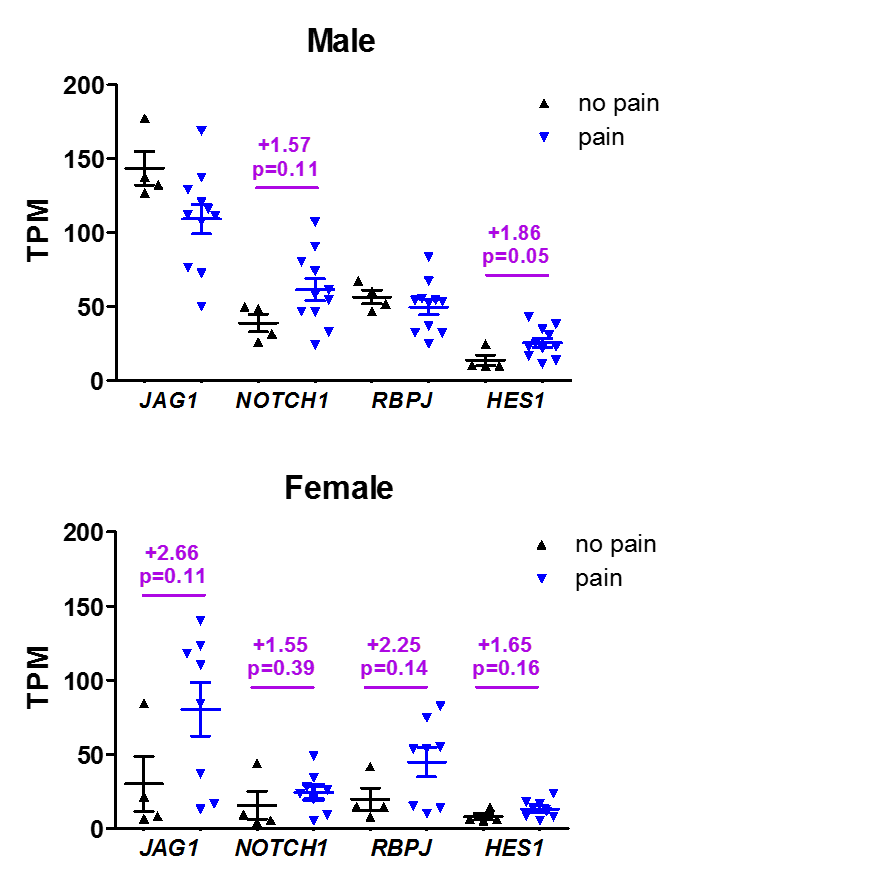
**

**Supp. Fig. 4.** Notch signaling genes (JAG1, NOTCH1, RBPJ, and HES1) in human DRG. Raw data of TPM (transcripts per million) were obtained from comparative transcriptome profile analysis of L2 lumbar DRG of human donors ([34](#_ENREF_34), [35](#_ENREF_35)). Gene expression was compared between donors without pain and those with neuropathic pain using unpaired 2-tailed Student's t-test. Totally 15 males (4 “no pain” and 11 “pain”) and 12 females (4 “no pain” and 8 “pain”) were analyzed.
